# New York

**Published:** 1896-04

**Authors:** 


### CONTROL WORK.

*New York State.* Important step in the Empire State. Preamble and resolutions relating to the importance of efficient measures for the suppression of tuberculosis in dairy-cattle, adopted by the Conference Committee of the State Board of Health of New York and the Board of Health of New York City.

*Whereas*, Tuberculosis is the most common and fatal disease occurring among the inhabitants of New York State and New York City; and, *Whereas*, Tuberculosis has been shown to be an infectious disease, and transmitted solely through direct communication from diseased animals or man to the healthy; and, *Whereas*, Tuberculosis has been proven by the investigations of the Tuberculosis Commission of the State Board of Health to be very prevalent among dairy-cattle in New York State; and, *Whereas*, There is abundant evidence that the milk of cows suffering from tuberculosis of different organs of the body, and particularly from tuberculosis of the mammary glands, frequently contains tubercle bacilli and is capable of producing tuberculosis in animals fed with it; and, *Whereas*, There is every reason to believe that tuberculosis in human beings, and especially in invalids, infants, and young children, is frequently caused by drinking the milk from tuberculous cows; and, *Whereas*, Experience has shown that it is possible with certainty to detect the existence of tuberculosis in cattle by the use of the tuberculin-test; and, *Whereas*, In the opinion of this Committee no subject is of greater sanitary importance than that pertaining to the suppression of tuberculosis in men and animals; and, *Whereas*, The reports of the work of the Tuberculosis Commission and the Tuberculosis Committee of the State Board of Health, made under the special appropriation for the investigation of the subject by the Legislature in 1893, 1894, and 1895, have proven and have shown that it has been successful within the limits set by meagre appropriations, and have clearly demonstrated, as has the experience in other States, that half-way measures cannot lead to the eradication of this disease, and have emphasized the absolute necessity for far more liberal appropriations for this purpose; therefore, be it *Resolved*, That, in order to secure the result already obtained and continue the work for the current fiscal year, such appropriation should, in the opinion of this Committee, be not less than $300,000.

Signed by Daniel Lewis, President New York State Board of Health; A. H. Doty, Health Officer, Port of New York; Owen Cassidy, Member of New State Board of Health; Charles G. Wilson, President of New York City Health Board; George B. Fowler, Commissioner of Health, New York City; T. Mitchell Prudden, Consulting Bacteriologist, Health Department, New York City; Hermann M. Biggs, Director Bacteriological Laboratory, Health Department, New York City.

"Dangerous, if true." The disposal of the famous Hawley herd of cattle to many parts of the United States, said to have taken place because the owner feared that tuberculosis would be found among others and their condemnation yield him not ten cents on a dollar of their worth, is a serious matter; and if any of these cattle were diseased, and are allowed to pass to other States and countries to spread tuberculosis, it lays a very heavy responsibility upon some of New York State's officers, and the end of this trouble may have only begun for all concerned. No State can knowingly allow any diseased animal to go to another State, from which disease and loss may become widespread as a result thereof, without being responsible.

